# Comparison of two T-cell assays to evaluate T-cell responses to SARS-CoV-2 following vaccination in naïve and convalescent healthcare workers

**DOI:** 10.1093/cei/uxac042

**Published:** 2022-05-06

**Authors:** Eloise Phillips, Sandra Adele, Tom Malone, Alexandra Deeks, Lizzie Stafford, Susan L Dobson, Ali Amini, Donal Skelly, David Eyre, Katie Jeffery, Christopher P Conlon, Christina Dold, Ashley Otter, Silvia D’Arcangelo, Lance Turtle, PITCH Consortium, Eleanor Barnes, Jeremy Chalk, Susanna Dunachie, Christopher Duncan, Paul Klenerman, Philippa Matthews, Rebecca Payne, Alex Richter, Thushan de Silva, Sarah Rowland-Jones, Lance Turtle, Dan Wootton, Paul Klenerman, Eleanor Barnes, Susanna J Dunachie

**Affiliations:** Peter Medawar Building for Pathogen Research, Nuffield Department of Clinical Medicine, University of Oxford, Oxford, UK; Peter Medawar Building for Pathogen Research, Nuffield Department of Clinical Medicine, University of Oxford, Oxford, UK; Peter Medawar Building for Pathogen Research, Nuffield Department of Clinical Medicine, University of Oxford, Oxford, UK; Peter Medawar Building for Pathogen Research, Nuffield Department of Clinical Medicine, University of Oxford, Oxford, UK; Oxford University Hospitals NHS Foundation Trust, John Radcliffe Hospital, Oxford, UK; Oxford University Hospitals NHS Foundation Trust, John Radcliffe Hospital, Oxford, UK; NIHR Health Protection Research Unit in Emerging and Zoonotic Infections, Institute of Infection, Veterinary and Ecological Sciences, University of Liverpool, UK; Oxford University Hospitals NHS Foundation Trust, John Radcliffe Hospital, Oxford, UK; Translational Gastroenterology Unit, University of Oxford, Oxford, UK; Oxford University Hospitals NHS Foundation Trust, John Radcliffe Hospital, Oxford, UK; Nuffield Department of Clinical Neuroscience, University of Oxford, UK; Oxford University Hospitals NHS Foundation Trust, John Radcliffe Hospital, Oxford, UK; Big Data Institute, University of Oxford, Oxford, UK; Oxford University Hospitals NHS Foundation Trust, John Radcliffe Hospital, Oxford, UK; Radcliffe Department of Medicine, University of Oxford, Oxford, UK; Oxford University Hospitals NHS Foundation Trust, John Radcliffe Hospital, Oxford, UK; Oxford Centre for Global Health Research, Nuffield Department of Clinical Medicine, University of Oxford, Oxford, UK; Oxford Vaccine Group, Department of Paediatrics, University of Oxford, Oxford, UK; NIHR Oxford Biomedical Research Centre, University of Oxford, Oxford, UK; UK Health Security Agency, Porton Down, UK; UK Health Security Agency, Porton Down, UK; NIHR Health Protection Research Unit in Emerging and Zoonotic Infections, Institute of Infection, Veterinary and Ecological Sciences, University of Liverpool, UK; Tropical and Infectious Disease Unit, Liverpool University Hospitals NHS Foundation Trust, member of Liverpool Health Partners, Liverpool, UK; University of Oxford; University of Oxford; University of Oxford; Newcastle University; University of Oxford; University of Oxford; Newcastle University; University of Birmingham; University of Sheffield; University of Sheffield; University of Liverpool; University of Liverpool; Peter Medawar Building for Pathogen Research, Nuffield Department of Clinical Medicine, University of Oxford, Oxford, UK; Oxford University Hospitals NHS Foundation Trust, John Radcliffe Hospital, Oxford, UK; Translational Gastroenterology Unit, University of Oxford, Oxford, UK; NIHR Oxford Biomedical Research Centre, University of Oxford, Oxford, UK; Peter Medawar Building for Pathogen Research, Nuffield Department of Clinical Medicine, University of Oxford, Oxford, UK; Oxford University Hospitals NHS Foundation Trust, John Radcliffe Hospital, Oxford, UK; Translational Gastroenterology Unit, University of Oxford, Oxford, UK; NIHR Oxford Biomedical Research Centre, University of Oxford, Oxford, UK; Peter Medawar Building for Pathogen Research, Nuffield Department of Clinical Medicine, University of Oxford, Oxford, UK; Oxford University Hospitals NHS Foundation Trust, John Radcliffe Hospital, Oxford, UK; Oxford Centre for Global Health Research, Nuffield Department of Clinical Medicine, University of Oxford, Oxford, UK; Mahidol-Oxford Tropical Medicine Research Unit, Bangkok, Thailand

**Keywords:** T cell, SARS-CoV-2, vaccination, infection, virus

## Abstract

T-cell responses to SARS-CoV-2 following infection and vaccination are less characterized than antibody responses, due to a more complex experimental pathway. We measured T-cell responses in 108 healthcare workers (HCWs) using the commercialized Oxford Immunotec T-SPOT Discovery SARS-CoV-2 assay service (OI T-SPOT) and the PITCH ELISpot protocol established for academic research settings. Both assays detected T-cell responses to SARS-CoV-2 spike, membrane, and nucleocapsid proteins. Responses were significantly lower when reported by OI T-SPOT than by PITCH ELISpot. Four weeks after two doses of either Pfizer/BioNTech BNT162b or ChAdOx1 nCoV-19 AZD1222 vaccine, the responder rate was 63% for OI T-SPOT Panels 1 + 2 (peptides representing SARS-CoV-2 spike protein excluding regions present in seasonal coronaviruses), 69% for OI T-SPOT Panel 14 (peptides representing the entire SARS-CoV-2 spike), and 94% for the PITCH ELISpot total spike. The two OI T-SPOT panels correlated strongly with each other showing that either readout quantifies spike-specific T-cell responses, although the correlation between the OI T-SPOT panels and the PITCH ELISpot total spike was moderate. The standardization, relative scalability, and longer interval between blood acquisition and processing are advantages of the commercial OI T-SPOT assay. However, the OI T-SPOT assay measures T-cell responses at a significantly lower magnitude compared to the PITCH ELISpot assay, detecting T-cell responses in a lower proportion of vaccinees. This has implications for the reporting of low-level T-cell responses that may be observed in patient populations and for the assessment of T-cell durability after vaccination.

## Teaser

T cells are important in protection against viral infections, but measuring T-cell responses is more complex than measuring antibodies. We measured T-cell responses in 108 healthcare workers to compare two assays, namely, the commercialized Oxford Immunotec T-SPOT Discovery SARS-CoV-2 assay (OI T-SPOT) and the PITCH ELISpot protocol established for academic research settings. Both assays detected T-cell responses to SARS-CoV-2 spike, M and NP proteins, but responses were significantly lower when reported by OI T-SPOT than by PITCH ELISpot.

## Graphical Abstract

**Figure:** In vaccinated healthcare workers the commercial Oxford Immunotec T-SPOT Discovery SARS-CoV-2 assay detects T cell responses to SARS-CoV-2 spike (Panel 1 + 2 and Panel 14), but at a lower magnitude than is reported by our in house PITCH ELISpot assay (total spike)

## Introduction

With the rapid roll-out of SARS-CoV-2 vaccinations across global healthcare systems, measurement of immune responses in both partially and fully vaccinated individuals is desirable for comparison of vaccination regimens, evaluation of immunocompromised individuals, monitoring responses to emerging variants of concern, and for determining the need for boosters. Surveillance of immune responses can guide COVID-19 vaccine rollout schemes to reduce the risk of infection and disease severity, and enable better allocation of healthcare resources. A number of cross-sectional and prospective cohort studies of healthcare workers (HCWs) have been established to monitor immune responses in individuals with vaccine- and/or infection-acquired immunity [1–3].

Monitoring of antibody responses to SARS-CoV-2, especially neutralizing antibodies, receives the most focus from researchers and policy makers. Serum for antibody assays is relatively easy to collect and store, binding antibodies can be measured at scale by automated platforms, and correlations with protection at a population level have been observed for binding [4] and neutralizing antibodies [5–8]. Nevertheless, T cells are a key arm of the immune response, orchestrating the antigen-specific adaptive response to infection including optimal production of antibodies from B cells, as well as having cytotoxic properties against virally infected cells. There is some evidence in macaque models and humans that the T-cell response is important in defence against SARS-CoV-2 [9, 10]. T-cell responses are maintained after boosting with a second vaccine dose [3], and the anti-spike (anti-S) T-cell response following vaccination with Pfizer/BNT162b2 does not correlate precisely with anti-S IgG antibody response [3]. Importantly, unlike the humoral response, the T-cell response to SARS-CoV-2 is minimally impacted by mutations in the α, β, γ, and λ variants of concern [3, 11], and 75–85% preserved against the omicron variant [12–18]. Therefore quantifying the T-cell response to SARS-CoV-2 is important but such monitoring is largely restricted to dedicated research centers due the technical expertise required to isolate cells from fresh blood within hours of blood draw, and the relative complexity of assays.

The *ex vivo* interferon-γ enzyme-linked absorbent spot (IFN-γ ELISpot) assay is a common workhorse assay used to measure antigen-specific T-cell responses. Specifically, ELISpot measures secreted cytokines at the single-cell level from peripheral blood, and by stimulating these cells with specific antigens of interest, T-cell responses to these antigens can be monitored. The main advantage afforded by ELISpot is its sensitivity, which exceeds intracellular cytokine staining (ICS) assays [19] and is up to 200 times greater than ELISA [20]. The assay takes 2-days to perform using skilled technicians. For this reason, collaboration with third-party diagnostic companies is becoming increasingly common in clinical research, especially for SARS-CoV-2. Such collaborations enable efficient data output and facilitate rapid research, the results of which can accordingly be used to inform clinical practice and patient management.

The PITCH (Protective Immunity from T-cells in Healthcare workers) study is a UK multi-centre prospective, observational cohort study in Oxford, Birmingham, Liverpool, Newcastle, and Sheffield which investigates T-cell responses in both vaccine- and/or infection-acquired immunity to SARS-CoV-2 infection [21]. We used this setting to evaluate the use of the proprietary Oxford Immunotec T-SPOT Discovery SARS-CoV-2 assay service [22] (OI T-SPOT), alongside T-cell measurement by our in-house IFN-γ ELISpot assay using the PITCH protocol, that has been harmonized across the five PITCH centers.

This study sought to compare the use of the Oxford Immunotec T-SPOT Discovery SARS-CoV-2 assay (OI T-SPOT) in reporting T-cell responses specific to SARS-CoV-2 spike and structural proteins, with our in-house PITCH ELISpot, from the same blood draw. Both assays are based on the ELISpot technique. The OI T-SPOT assay, which is an example of a commercial interferon-γ release assay (IGRA), was introduced by Oxford Immunotec 15 years ago as a diagnostic test for *Mycobacterium tuberculosis* (T-SPOT.*TB* test). A kit containing pre-coated plates and anti-IFN-γ antibodies is available for immunology laboratories. This initial technology was optimized to enumerate *M. tuberculosis*-sensitized T cells by measuring IFN-γ secreted from CD4 and CD8 T cells in response to antigens from *M. tuberculosis* (reviewed [23]). As the SARS-CoV-2 pandemic spread, this technology was subsequently adapted to allow SARS-CoV-2-specific T cells to be enumerated and is currently available as a service requiring shipping of fresh blood to Oxford Immunotec, Abingdon, UK. The PITCH ELISpot protocol has origins at the Jenner Institute [24] where it was initially developed to enumerate T-cell responses to malaria by measuring IFN-γ secretion to a pre-erythrocytic malaria antigen, thrombospondin-related adhesion protein (TRAP). This technique was later optimized for SARS-CoV-2 antigens [25] before being harmonized for PITCH across five UK laboratories [21]. Here we compared these two SARS-CoV-2 ELISpot assays from the same blood draw to assess the utility of the OI T-SPOT assay.

## Materials and methods

### Study design and participants

In this prospective, observational, cohort study, we sampled participants at one PITCH center in Oxford. HCWs were enrolled in the OPTIC study (GI Biobank Study 16/YH/0247, approved by the research ethics committee (REC) at Yorkshire & The Humber—Sheffield Research Ethics Committee on 29 July 2016, and amended for the OPTIC study on 8 June 2020). Healthy men and women aged 18 years and older were recruited.

Previous SARS-CoV-2 infection status was defined in HCWs based on documented PCR and/or baseline anti-S and anti-N serology results from the Abbott platform at Oxford University Hospital NHS Foundation Trust prior to vaccination. All participants received either the BNT162b Pfizer/BioNTech vaccine or the ChAdOx1 nCoV-19 AZD1222 vaccine.

### Oxford Immunotec T-SPOT discovery SARS-CoV-2 assay

A total of 6 ml of sodium heparinized whole blood per participant was shipped to Oxford Immunotec, Abingdon, UK under a commercial contract. Oxford Immunotec is independent of the University of Oxford. Samples were typically shipped the same day with a few samples sent after overnight storage but within 32 h of blood draw. According to OI information, peripheral blood mononuclear cells (PBMCs) were isolated from the whole blood and 250 000 PBMCs were added per well in the T-SPOT Discovery SARS-CoV-2 kit, an ELISPOT assay modified to measure SARS-CoV-2-specific T-cell responses [26]. Each well contains an optimized antigen pool, including SARS-CoV-2 structural proteins, which stimulates T cells *in vitro* allowing their response to individual SARS-CoV-2 proteins to be measured. Peptide regions of high homology to other endemic coronaviruses were removed. Alongside negative (nil control) and positive controls (phytohaemagglutinin–PHA, a known polyclonal activator), a total of five SARS-CoV-2 pools are used; S1 diagnostic (Panel 1), S2 diagnostic (Panel 2), M, NP and total spike (Panel 14), ensuring the maximal breadth of the T-cell response is investigated. Sequence details of the peptides were undisclosed. The background (nil control) was subtracted from test pools of interest and responses were multiplied by four to report here as spot-forming units (SFU) per 10^6^ peripheral blood mononuclear cells (PBMCs).

### In-house PITCH ELISpot

The PITCH frozen ELISpot standard operating procedure has been published previously [21] and has been optimized for cryopreserved PBMC for easier workflow across laboratories, although higher responses are achieved using fresh blood [21]. PBMCs isolated from the same blood draw as the OI T-SPOT assay were cryopreserved in fetal bovine serum and 10% DMSO and later were thawed in a water bath at 37^o^C and added dropwise to 9 ml R0 (RPMI media supplemented with L-Glutamine and 10 mM penicillin/streptomycin) at room temperature (RT) and then centrifuged at 400*g* for 5 min. Cell pellets were then resuspended and washed in 5 ml of Rab10 (filtered R0 supplemented with 10% human serum) at RT and centrifuged again. These pellets were then resuspended in 5 ml Rab10 supplemented with DNase and allowed to rest for 2–3 h in an incubator at 37^o^C, 5% CO_2_, and 95% humidity. Interferon-γ (IFN-γ) ELISpot assays were performed using the Human IFN-γ ELISpot Basic kit (Mabtech 3420-2A). MultiScreen-I 96 ELISpot plates (Millipore, MAIPS4510) were coated overnight at 4^o^C or for 3–8 h at RT with the capture antibody (clone 1-D1K) at 5 ug/ml in PBS. Coated plates were subsequently washed twice with R0 and then blocked with 100 ul/well of Rab10 for 1/2–8h at RT or 8–48 h at 4^o^C. Rested cells were centrifuged and resuspended in 1 ml Rab10 for counting on Muse^TM^ Cell Analyser or Bio-Rad TC10^TM^ Automated Cell Counter. After blocking, overlapping peptide pools (18-mers with 10 amino acid overlap, Mimotopes) representing spike (S1, S2), membrane (M), and nucleocapsid (NP) SARS-CoV-2 proteins were added to 200 000 PBMCs/well at a final concentration of 2 ug/ml for 16–18 h. S1 and S2 were added in separate test wells, M and NP were combined in a singular test well. Assays were performed in triplicate. EBV, influenza, and tetanus toxoid peptide pools (2 ug/ml, Proimmune PX-CEFT peptide pool) and concanavalin A (5 ug/ml) (ConA) were used as positive controls, along with negative control wells (DMSO in Rab10). After overnight peptide stimulation, plates were washed seven times with 100–200 ul/well PBS–0.05% Tween and then incubated for 2–4 h at RT with 50 ul/well of 1 ug/ml biotinylated detection antibody (clone 7-B6-1) diluted in PBS. Plates were then washed again as above and incubated for 1–2 h with 50 ul/well of 1 ug/ml streptavidin-ALP diluted in PBS. After a final wash, spots were detected by adding 50 ul/well of filtered RT BCIP/NBT stock and incubating for 5 min in the dark. Colour development was stopped by the removal of BCIP and rinsing with cold water. Plates were air-dried for at least two nights and subsequently read on the CTL immunocapture (Cellular Technology Limited, Shaker Heights, Ohio, USA) using the Smartcount® settings. The mean spots of the negative control wells were subtracted from the test wells and then multiplied by five to give antigen-specific responses expressed as spot-forming units (SFU) per 10^6^ PBMCs. Total spike responses were defined by adding S1 and S2 responses together. The PITCH protocol as described uses 3.2 million PBMC.

### Serological assays

Anti-spike (S) and anti-nucleocapsid (N) antibodies were measured using the Roche Elecsys® Anti-SARS-CoV-2 S and Roche Elecsys® Anti-SARS-CoV-2 N assays at the Public Health England (now the United Kingdom Health Security Agency) Laboratories at Porton Down, UK. The Roche S assay is reported in units per milliliter (U/ml), which are standardized 1:1 to the WHO binding antibody units/ml (BAU/ml). Seroconversion is defined for S as a response equal to or greater than 0.8 U/ml, and for N as a response equal to or greater than 1.0 COI.

### Statistical analysis

Data were analyzed in GraphPad Prism 9.1.2. Non-parametric tests were used to assess significance between data sets as non-Gaussian distribution was assumed. For matched samples, Wilcoxon’s test was used to compare two groups and Friedman’s test was used to compare three or more groups, accounting for multiple comparisons. For unmatched samples, Mann–Whitney test was used to compare two groups and Kruskal–Wallis test was used to compare three or more groups. For analyzing correlations, two-tailed non-parametric Spearman correlation was performed. Two-tailed *P* values were reported with less than 0.05 considered significant.

## Results

### Human participants

A total of 108 participants, including both SARS-CoV-2 infection-naïve (*n =* 83) and previously infected (*n* = 25) HCWs in Oxford, UK, were included in the study where matched data from OI T-SPOT and the PITCH ELISpot assays were available. Participants were sampled just before their second dose of vaccine which was a median of 9.86, interquartile range (IQR) 6.6–11 weeks after their first dose (1 dose + 10 weeks), and again a median of 4.3, IQR 4–4.6 weeks after the second dose (2 doses + 4 weeks). Pre-vaccination samples at baseline were available for a limited number of participants, but without matched results for OI T-SPOT and the PITCH ELISpot assays. All sampling was between December 2020 and July 2021. Demographic details of the participants are shown in Table 1. Anti-S and anti-N binding antibodies measured by Roche are shown in Supplementary Fig. S1, with all participants seroconverting to anti-spike positivity 4 weeks after the second dose of vaccine (range 450–42 510 AU/ml).

**Table 1:** Characteristics of healthcare workers included in the study.

	All	Naïve	Previously infected
Dosing interval			
Days, median (IQR)	69 (46–77)	69 (36.5–77)	66 (63–77)
Days, range	17–92	17–92	19–90
Weeks, median	9.86	9.86	9.43
Days post V2			
Median (IQR)	30 (28–32)	30 (28–32.8)	29.5 (26–31.3)
*N*	108	83	25
Female, *N* (%)	68 (63%)	48 (58%)	20 (80%)
Male, *N* (%)	40 (37%)	35 (42%)	5 (20%)
Mean age	35.14	34.74	36.44
Age in years, median (IQR)	33 (24–42.5)	33 (23–42.8)	35 (28–42)
Age range	21–66	21–66	21–63
Infection status, *N* (%)			
Naïve	83 (77%)	83 (100%)	0 (0%)
Previous SARS-CoV-2	25 (23%)	0 (0%)	25 (100%)
Ethnicity (self-reported*), *N* (%)			
White*	90 (83%)	68 (82%)	22 (88%)
Asian*	10 (9%)	8 (10%)	2 (8%)
Black*	0 (0%)	0 (0%)	0 (0%)
Other*	5 (5%)	4 (5%)	1 (4%)
Unreported	3 (3%)	3 (4%)	0 (0%)

T-cell responses to SARS-CoV-2 spike and structural proteins were measured by commercialized OI T-SPOT and in-house PITCH ELISpot

**Figure 1:**
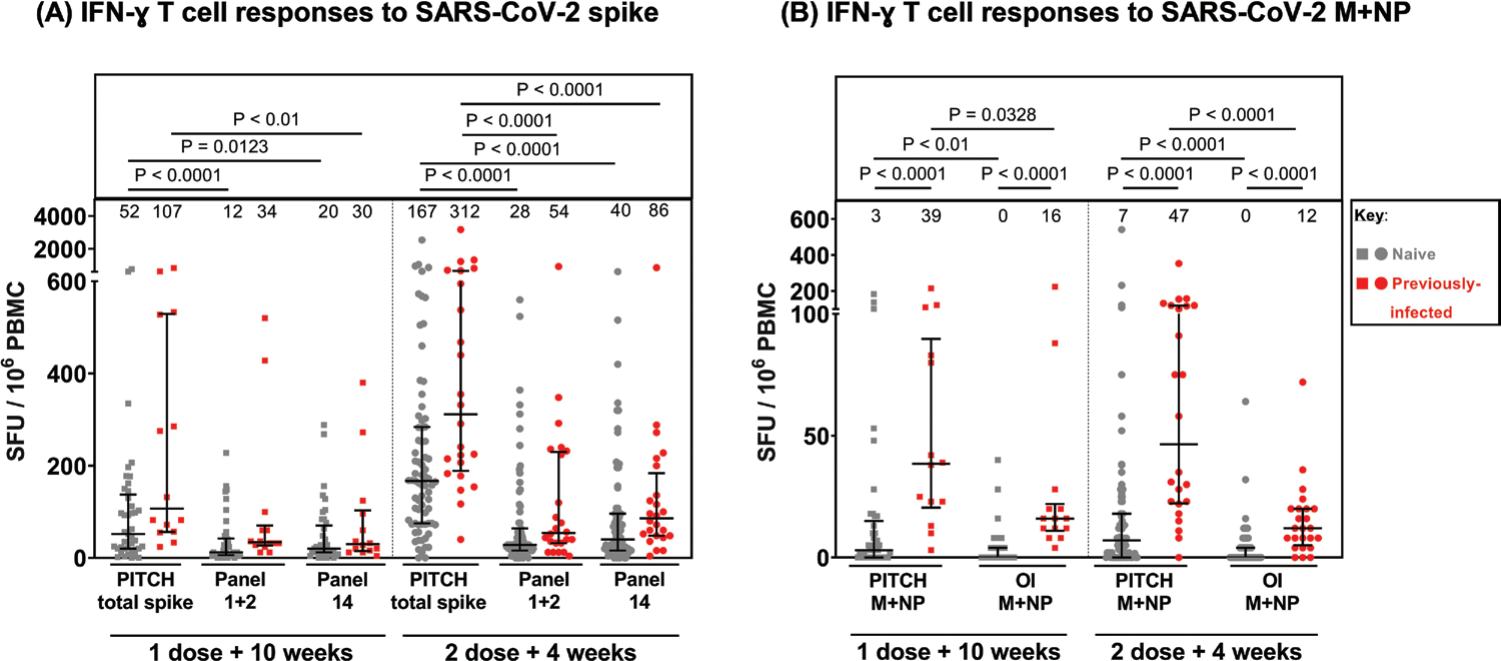
Comparison of T-cell responses to SARS-CoV-2 spike and structural proteins measured by in-house PITCH ELISpot and Oxford Immunotec T-SPOT assay. (**A**) T-cell responses to SARS-CoV-2 spike in naïve and previously infected healthcare workers reported by three panels: PITCH total spike (S1+S2), Oxford Immunotec Panel 1 + 2 (diagnostic S1 + S2), and Oxford Immunotec Panel 14 (total spike). Friedman test was used for statistical analysis between the three panels. (**B**) T-cell responses to SARS-CoV-2 membrane (M) and nucleocapsid (NP) in naïve and previously infected healthcare workers reported by PITCH ELISpot and Oxford Immunotec (OI) T-SPOT assay. Wilcoxon test was used for statistical analysis between samples matched across both assays, and Mann–Whitney test was used to compare naïve and previously infected T-cell responses within subgroups. (**A,B**) T-cell responses are quantified by spot-forming units (SFU) per 10^6^ peripheral blood mononuclear cells (PBMCs). Healthcare workers received phlebotomy 10 weeks post 1st dose (1 dose + 10 weeks) and/or 4 weeks post 2nd dose (2 doses + 4 weeks). All samples are matched across both assays (three panels for spike and 2 for M + NP T-cell responses). At 1 dose + 10 weeks, *n* = 41 for naïve samples and *n* = 14 for previously infected samples. At 2 doses + 4 weeks, *n* = 75 for naïve samples and *n* = 24 for previously infected samples. Infection status at time of first vaccine, as defined by available PCR and serology data: grey symbols = naïve HCWs and red symbols = HCWs previously infected with SARS-CoV-2. Median T-cell responses are stated immediately above each column and marked by a horizontal line on each column, and interquartile range is represented by error bars.

T-cell responses to spike antigens after vaccination were detected by both OI T-SPOT and PITCH ELISpot assays (Fig. 1A; Supplementary Table S1), with higher responses recorded by the PITCH assay. At 2 doses + 4 weeks, median spike-specific T-cell responses in the naïve cohort measured by OI Panel 1 + 2 and OI Panel 14 were 28 (IQR 16–64) and 40 (IQR 16–96) SFU/10^6^ PBMCs, respectively, 6.0- and 4.2-fold lower than PITCH total spike (median = 167, IQR 75–284 SFU/10^6^ PBMCs; *P* < 0.0001). Median responses were numerically lower for the OI Panel 1 + 2 (where peptides representing regions in the spike protein of high homology to other endemic coronaviruses had been removed) compared to OI Panel 14 (peptides representing the full spike protein) although there was no statistically significant difference between the two.

In SARS-CoV-2 infection-naïve HCWs at dose 2 + 4 weeks, the median T-cell response to SARS-CoV-2 M+NP is 0 (IQR 0–4) SFU/10^6^ PBMCs in OI T-SPOT and 7 (IQR 0–18) SFU/10^6^ PBMCs in PITCH ELISpot (*P* < 0.0001, Fig. 1B). As expected, both assays report significantly higher responses in previously infected cohorts compared to naïve samples at each timepoint (*P* < 0.0001). Only previously infected participants are expected to have T-cell responses specific to SARS-CoV-2 structural proteins as natural infection involves exposure to the whole SARS-CoV-2 proteome including M + NP. Vaccination with either BNT162b Pfizer/BioNTech or ChAdOx1 nCoV-19 AZD1222 involves exposure to only SARS-CoV-2 spike protein, so naïve participants should be negative for T-cell responses to SARS-CoV-2 non-spike proteins. However, some participants characterized as “naïve” may have been exposed to SARS-CoV-2 without symptoms or seroconversion. Overall, our findings support the use of both assays for identifying differences in these responses between cohorts based on infection status.

### Correlation between SARS-CoV-2-specific T-cell responses measured by commercialized OI T-SPOT and in-house PITCH ELISpot

**Figure 2:**
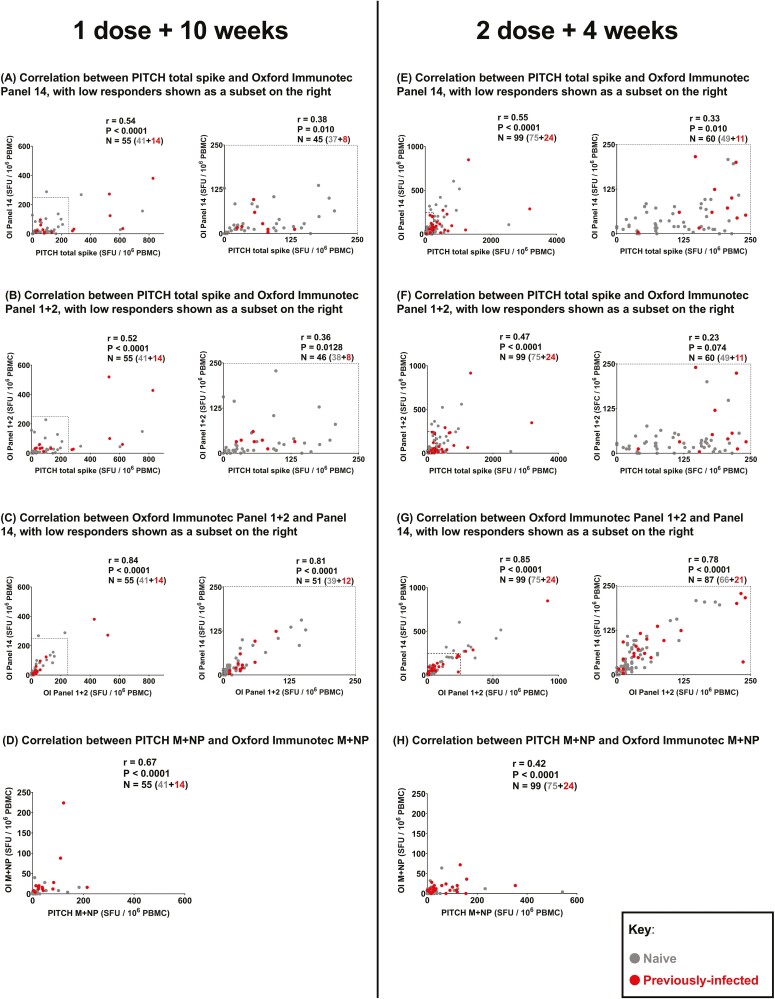
Correlation between T-cell responses to SARS-CoV-2 spike and structural proteins measured by in-house PITCH ELISpot and OI T-SPOT assay. (**A**–**C**) Correlation between T-cell responses to SARS-CoV-2 spike protein measured at 1 dose + 10 weeks timepoint by three different panels: PITCH total spike, Oxford Immunotec Panel 1 + 2 and Oxford Immunotec Panel 14. *n* = 55 (*n* = 41 naïve, *n* = 14 previously infected). (**A**) Correlation between PITCH total spike and Oxford Immunotec Panel 14 at 1 dose + 10 weeks, with low responders (≤250 SFC/10^6^ PBMC) delineated by a dotted-line in the graph on the left and represented in the graph on the right (*n* = 45 total; *n* = 37 naïve and *n* = 8 previously-infected). (B) Correlation between PITCH total spike and Oxford Immunotec Panel 1 + 2 at 1 dose + 10 weeks, with low responders (≤250 SFC/10^6^ PBMC) delineated by a dotted-line in the graph on the left and represented in the graph on the right (*n* = 46 total; *n* = 38 naïve and *n* = 8 previously-infected). (**C**) Correlation between Oxford Immunotec Panel 1 + 2 and Panel 14 at 1 dose + 10 weeks, with low responders (≤250 SFC/10^6^ PBMC) delineated by a dotted-line in the graph on the left and represented in the graph on the right (*n* = 51 total; *n* = 39 naïve, *n* = 12 previously-infected). (**D**) Correlation between T-cell responses to SARS-CoV-2 membrane and nucleocapsid (M + NP) protein at 1 dose + 10 weeks timepoint measured by PITCH ELISpot and Oxford Immunotec T-SPOT. *n* = 55 (*n* = 41 naïve, *n* = 14 previously infected). (**E**–**G**) Correlation between T-cell responses to SARS-CoV-2 spike protein measured at 2 doses + 4 weeks timepoint by three different panels: PITCH total spike, Oxford Immunotec Panel 1 + 2 and Oxford Immunotec Panel 14. *n* = 99 (*n* = 75 naïve, *n* = 24 previously infected). (**E**) Correlation between PITCH total spike and Oxford Immunotec Panel 14 at 2 doses + 4 weeks, with low responders (≤250 SFC/10^6^ PBMC) delineated by a dotted-line in the graph on the left and represented in the graph on the right (*n* = 60 total; *n* = 49 naïve and *n* = 11 previously infected). (**F**) Correlation between PITCH total spike and Oxford Immunotec Panel 1 + 2 at 2 doses + 4 weeks, with low responders (≤250 SFC/10^6^ PBMC) delineated by a dotted-line in the graph on the left and represented in the graph on the right (*n* = 60 total; *n* = 49 naïve and *n* = 11 previously infected). (**G**) Correlation between Oxford Immunotec Panel 1 + 2 and Panel 14 at 2 doses + 4 weeks, with low responders (≤250 SFC/10^6^ PBMC) delineated by a dotted-line in the graph on the left and represented in the graph on the right (*n* = 87 total; *n* = 66 naïve, *n* = 21 previously infected). (H) Correlation between T-cell responses to SARS-CoV-2 membrane and nucleocapsid (M + NP) protein at 2 doses + 4 weeks timepoint measured by PITCH ELISpot and Oxford Immunotec T-SPOT. *n* = 99 (*n* = 75 naïve, *n* = 24 previously infected). (**A**–**H**) Spearman’s *r* correlation was performed and two-tailed *P* values reported (α = 0.05). Infection status at time of first vaccine, as defined by available PCR and serology data: grey symbols = naïve HCWs and red symbols = HCWs previously infected with SARS-CoV-2.

To further evaluate the use of OI T-SPOT, correlations between spike-specific T-cell responses reported by OI Panel 1 + 2, Panel 14, and PITCH total spike, were determined at 1 dose + 10 weeks (Fig. 2A–C) and 2 doses + 4 weeks (Fig. 2E–G). The observed correlation between OI spike panels (Panel 1 + 2 and Panel 14) and PITCH total spike is low to moderate at dose 1 + 10 weeks (Fig. 2A and B) and dose 2 + 4 weeks (Fig. 2E and F), with the strongest correlation being between OI Panel 14 and PITCH total spike at dose 2 + 4 weeks (*r* = 0.55, *P* < 0.0001, Fig. 2E) and the lowest between OI Panel 1 + 2 and PITCH total spike at dose 2 + 4 weeks (*r* = 0.47, *P* < 0.0001). As expected, Panel 1 + 2 and Panel 14 on OI correlate strongly at both timepoints (*r* = 0.84 and 0.85, respectively, *P* < 0.0001, Fig. 2C and G). Moreover, as correlations with PITCH total spike are comparable between OI Panel 1 + 2 and OI Panel 14, this suggests that either readout can be used to quantify T-cell responses to SARS-CoV-2 spike. Moderate correlations (*r* = 0.42 and 0.67, *P* < 0.0001) were observed for T-cell responses to M + NP reported by OI T-SPOT and PITCH ELISpot (Fig. 2D and H).

Since spike-specific T-cell responses measured by OI T-SPOT panels tended lower than those measured by PITCH ELISpot, we investigated whether the correlation between OI T-SPOT readouts (Panel 1 + 2 and Panel 14) and PITCH total spike changed when looking at low responders only. This subset was defined as having T-cell responses equal to or lower than 250 SFU/10^6^ PBMCs in both panels being correlated. At both 1 dose + 10 weeks (Fig. 2A–C) and 2 doses + 4 weeks (Fig. 2E–G), OI spike Panels (Panel 1 + 2 and Panel 14) and PITCH total spike weakly correlated with significant *r* values, ranging from *r* = 0.33 (*P* < 0.01, Fig. 2E) to *r* = 0.38 (*P* < 0.01, Fig. 2A). These correlations suggest that the PITCH ELISpot is more sensitive to detecting T-cell responses in low responders. However, overall OI T-SPOT valuably characterizes T-cell responses, which is an important component of COVID-19 research as T-cell and antibody responses do not always correlate (Supplementary Fig. S2).

### Defining positive responders to SARS-CoV-2 spike in both assays

We sought to investigate how positive responders could be numerically defined for each assay, and whether the proportion of positive responders differed across the three panels measuring SARS-CoV-2 spike-specific T-cell responses (Table 2). The OI T-SPOT assay uses test wells with cell concentrations equating to 250 000 PBMCs/well, while the PITCH ELISpot assay uses 200 000 PBMCs/well. We explored positivity as defined by a cut-off of 24 SFU/10^6^ PBMCs for the OI T-SPOT and 26 SFU/10^6^ PBMCs for the PITCH SOP ELISpot. OI provided the 24 SFU/10^6^ PBMCs cut-off (based on in-house research and development defining their cut-off as 6 SFU/250 000 PBMC) whereas we calculated the 26 SFU/10^6^ PBMCs PITCH SOP ELISpot cut-off as the mean of all negative control wells + 2 SD. Using these cut-offs, we find that the percentage of positive responders measured on OI Panel 1 + 2 and OI Panel 14 are comparable at 1 dose + 10 weeks (55% and 49%, respectively) and 2 doses + 4 weeks (63% and 69%), whereas PITCH total spike reports a higher proportion of positive responders at both 1 dose + 10 weeks (38/55, 69%) and at 2 doses + 4 weeks (93/99, 94%). The OI T-SPOT assay therefore detects T cells using this cut-off in a lower proportion than the PITCH ELISpot assay. Responder rates for the PITCH ELISpot assay were unaffected by calculating the cut-off separately for previously infected and infection-naïve participants.

**Table 2.** Table showing the number and percentage of positive responders to SARS-CoV-2 spike at 1 dose + 10 weeks and 2 doses + 4 weeks measured across three different panels: Oxford Immunotec Panel 1 + 2 and Panel 14 and PITCH total spike. Oxford Immunotec T-SPOT assay defines the cut-off for a positive responder as 6 SFU/250 000 PBMCs, which translates to 24 SFU/10^6^ PBMCs. The PITCH ELISpot assay defines the cut-off for a positive responder as 26 SFU/10^6^ PBMCs. This cut-off is calculated from negative control wells as: (mean + 2 SD). The cohort analyzed here is described in Table 1 (naïve and previously infected groups are combined).

	*n*	# Responders on Oxford immunotec Panel 1 + 2 (%)	# Responders on Oxford immunotec panel 14 (%)	# Responders on PITCH total spike (%)
Cut-off (SFU/10^6^ PBMCs)	NA	24	24	26
1 dose + 10 weeks	55	30 (55%)	27 (49%)	38 (69%)
2 doses + 4 weeks	99	62 (63%)	68 (69%)	93 (94%)

Baseline T-cell response measurements with either the OI T-SPOT assay or the PITCH ELISpot assay were available in a subset of HCWs only, without matched samples (Supplementary Fig. S3). We have previously established that the PITCH ELISpot assay is highly specific with no or minimal responses in pre-pandemic samples and in individuals early in the pandemic without exposure [25]. A few individuals identified as infection naïve (no history of positive PCR test and seronegative for anti-S and anti-N antibodies) at baseline prior to vaccination (December 2020 onwards) have T-cell responses to spike, and/or membrane and nucleocapsid proteins. HCWs were generally exposed to the virus in 2020, and these T-cell responses may represent undiagnosed SARS-CoV-2 infection in the absence of seroconversion as previously described [25, 27].

## Discussion

The cohort included both SARS-CoV-2-naïve and previously infected healthcare workers vaccinated with either BNT162b Pfizer/BioNTech or ChAdOx1 nCoV-19 AZD1222, and receiving phlebotomy prior to 2nd vaccine dose (1 dose + 10 weeks) or 4 weeks post 2nd dose (2 doses + 4 weeks). T-cell responses to SARS-CoV-2 spike, M, and NP proteins were lower when reported by OI Panels than by PITCH ELISpot, with the correlation between the assays. The OI T-SPOT assay appeared less effective at quantifying T-cell responses in low responders. As OI Panel 14 and OI Panel 1 + 2 correlated strongly in both entire cohorts and low responders, this suggests that either readouts may be used to quantify spike-specific T-cell responses. Similarly, the OI T-SPOT assay may also be used to quantify T-cell responses to SARS-CoV-2 M + NP as significant differences were reported between naïve and previously infected HCWs. Limitations of this study include the lack of assessment of standardizing positive controls and sample type as they differed between assays, with OI T-SPOT using PHA and fresh samples and PITCH ELISpot using ConA and frozen samples. However, if we had compared the OI T-SPOT with the PITCH ELISpot performed on fresh cells, this would have led to an even greater difference in magnitude between the two assays because a higher magnitude of antigen-specific IFN-γ responses is obtained from the PITCH ELISpot when performed on fresh cells versus frozen [21].

We have established the PITCH ELISpot protocol as a reproducible assay between experienced cellular immunology laboratories, with no significant responses seen in pre-pandemic archived samples nor in SARS-CoV-2 unexposed, seronegative donors early in the pandemic [28]. Thus, the PITCH ELISpot offers a useful gold standard method to quantify the T-cell response to SARS-CoV-2 infection and vaccines, with greater specificity than activation-induced marker (AIM) and proliferation assays and greater sensitivity than ICS. However, scaling up the PITCH ELISpot assay requires investment in training, equipment, and infrastructure which is not always possible for comparing large cohorts of clinical trials and patient populations.

The Oxford Immunotec T-SPOT discovery assay has been used to assess SARS-CoV-2 T-cell responses in a variety of UK research settings, particularly to gain an understanding of the T-cell response after vaccination. Prendecki *et al.* used the OI T-SPOT assay to monitor spike-specific T-cell responses in HCWs 3 weeks after the first dose of BNT162b Pfizer/BioNTech, importantly showing that previously infected HCWs had T-cell responses 10-fold higher than naïve HCWs [29]. Parry *et al.* similarly sought to characterize the spike-specific T-cell responses post-vaccination, instead 2 weeks after the second dose of BNT162b Pfizer/BioNTech and importantly in a cohort aged 80–96 years [30]. While cellular responses to spike were less common than antibody responses, 63% of people had detectable T-cell responses, demonstrating the utility of the OI T-SPOT assay in a cohort where T-cell responses might be lower due to immunosenescence [31]. In both these studies, the OI T-SPOT assay provided rapid and crucial insight into the cellular response, accompanying characterizations of the humoral response. These findings are important as antibody and T-cell responses do not always correlate [32] and the correlates of protection from SARS-CoV-2 remain to be determined. The OI T-SPOT assay provides the opportunity to study T-cell responses alongside antibody responses in the absence of a T-cell research laboratory, thus enabling the immune response to SARS-CoV-2 in different contexts to be determined. In addition, the OI T-SPOT assay has been utilized in large cohorts, for example, the Com-Cov-2 clinical trial, characterizing immune responses in participants vaccinated with different combinations of COVID-19 vaccines [33], and the UK OCTAVE study, which evaluates immune responses in vaccinated patients with immune-mediated inflammatory diseases, including cancer, inflammatory arthritis, diseases of the kidney or liver, or patients who are having a stem cell transplant [34].

## Conclusion

Here we show that the OI T-SPOT assay is a robust method of measuring T-cell responses to SARS-CoV-2 vaccination and infection, with particular benefits in national studies with relatively large cohorts. The OI T-SPOT assay offers the opportunity for evaluation at a relative scale not usually offered by T-cell research laboratories in the academic sector, and in settings where a research laboratory is not available. Additional benefits include the ability of OI to receive samples up to 32 h from blood draw (compared to 4 h for the PITCH protocol), the rapid turnaround time of results, which are received within a week of samples being dispatched to the laboratory, and standardization for comparing across different centers and studies. Disadvantages include the cost, the need to arrange transportation of fresh samples to the south of England, lower detection of T-cell responses compared to a research laboratory, and less flexibility to customize the assay as the pandemic evolves. Further evaluation is needed of the use of the OI assay in low responders, and further development to raise its sensitivity, for example increasing cell numbers, may enhance its utility in the study of immunocompromised patients with low antibody responses to the vaccines. Both assays share the disadvantage of requiring PBMC separation, and research continues by our laboratory and others to develop sensitive and specific whole blood T-cell assays. Antigen-specific interferon-γ responses are only one of many available measures of T-cell function. Overall, however, the OI T-SPOT assay offers an efficient and standardized approach for researchers for comparisons across vaccine platforms, dosing approaches, and research studies.

## Supplementary Material

uxac042_suppl_Supplementary_Figure_S1

uxac042_suppl_Supplementary_Figure_S2

uxac042_suppl_Supplementary_Figure_S3

uxac042_suppl_Supplementary_Table_S1

uxac042_suppl_Supplementary_Figure_Legends

## Data Availability

The data underlying this article are available in the article and in its online supplementary material.

## Abbreviations

AIM: activation-induced marker
ConA: concanavalin A
DMSO: dimethyl sulfoxide
ELISA: enzyme-linked immunosorbent assay
ELISpot: enzyme-linked immunosorbent spot
HCW: healthcare workers
ICS: intracellular cytokine staining
IFN-γ: Interferon-γ
IGRA: interferon-γ release assay
IQR: interquartile range
M+NP: membrane and nucleocapsid proteins
OI T-SPOT: Oxford Immunotec T-SPOT Discovery SARS-CoV-2 assay
OI: Oxford Immunotec
PBMCs: peripheral blood mononuclear cells
PBS: phosphate-buffered saline
PCR: polymerase chain reaction
PHA: phytohaemagglutinin
PITCH: Protective Immunity from T cells to Covid-19 in Health workers
REC: research ethics committee
RT: room temperature
SFU: spot forming units
SOP: standard operating procedure
TRAP: thrombospondin-related adhesion protein.
