# Supplementary figures and images for: Comparison of two T-cell assays to evaluate T-cell responses to SARS-CoV-2 following vaccination in naïve and convalescent healthcare workers
Source: Clin Exp Immunol. 2022 May 6;209(1):90–8. doi: 10.1093/cei/uxac042 (PMC9129206; doi:10.1093/cei/uxac042)

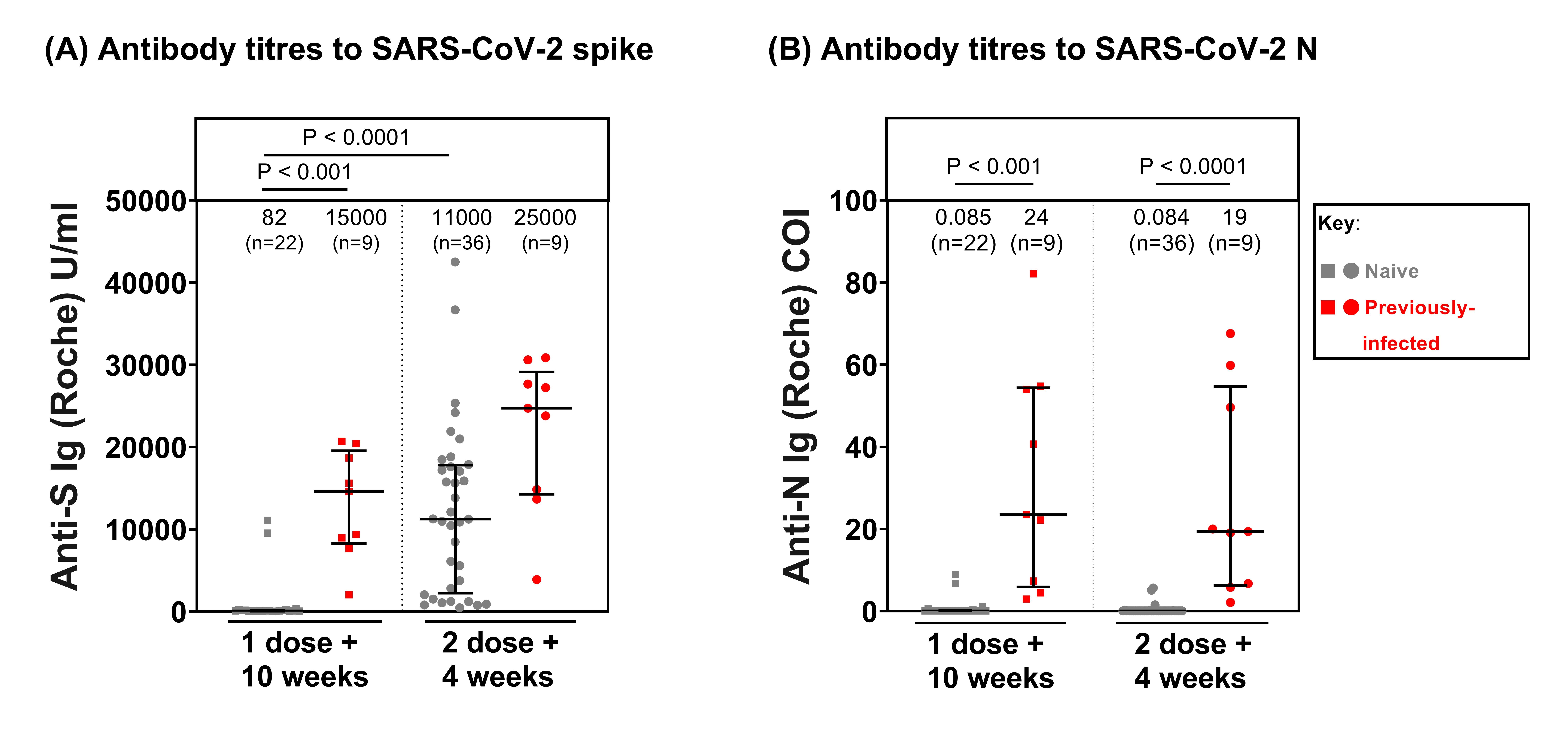

Supplement: uxac042_suppl_Supplementary_Figure_S1 [file uxac042_suppl_supplementary_figure_s1.jpeg]

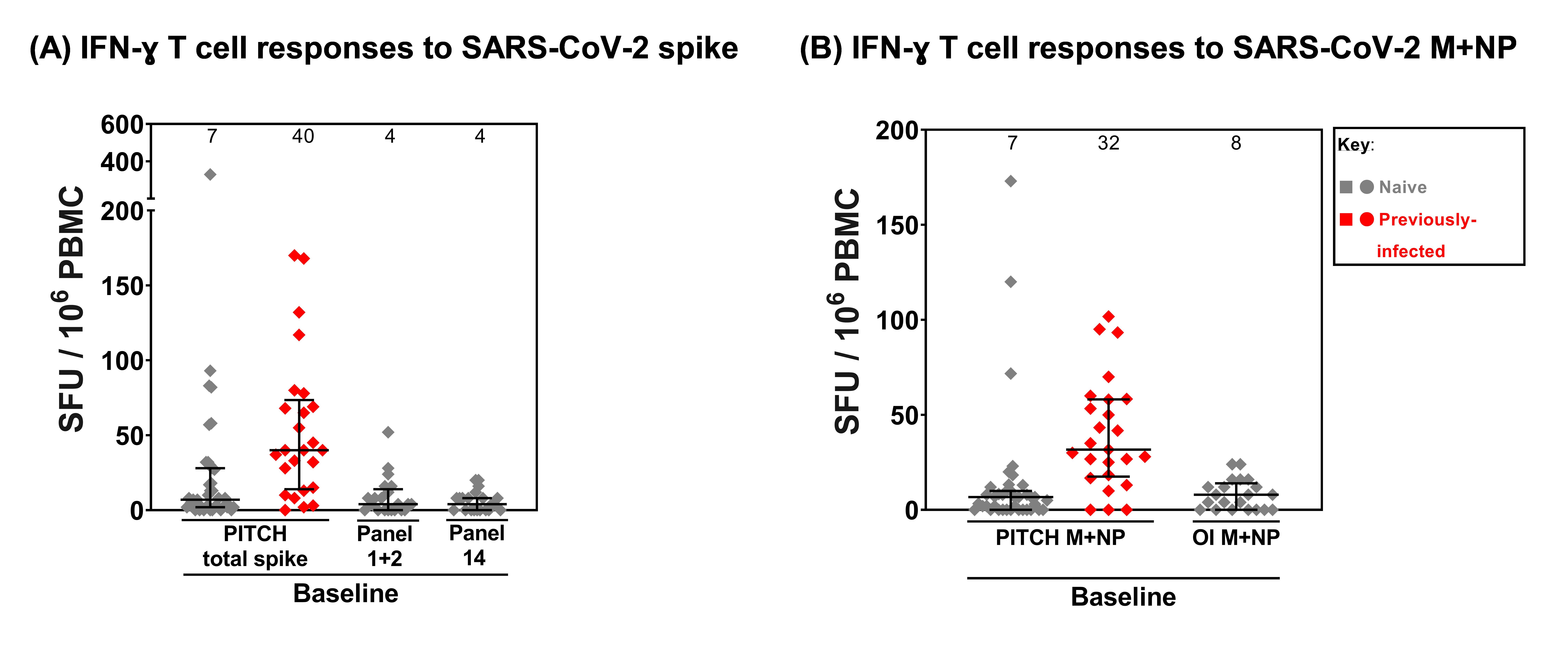

Supplement: uxac042_suppl_Supplementary_Figure_S3 [file uxac042_suppl_supplementary_figure_s3.jpeg]
