# Supplementary material for: Comparison of two T-cell assays to evaluate T-cell responses to SARS-CoV-2 following vaccination in naïve and convalescent healthcare workers
Source: Clin Exp Immunol. 2022 May 6;209(1):90–8. doi: 10.1093/cei/uxac042 (PMC9129206; doi:10.1093/cei/uxac042)
Supplement: uxac042_suppl_Supplementary_Table_S1 [file uxac042_suppl_supplementary_table_s1.docx]

|  | **1 dose + 10 weeks** | | **2 dose + 4 weeks** | |
| --- | --- | --- | --- | --- |
|  | **Naïve**  **(n=41)** | **Previously infected (n=14)** | **Naïve**  **(n=75)** | **Previously infected**  **(n=24)** |
| **Median (IQR)**  **(SFU/10^6^ PBMCs)** |  |  |  |  |
| **PITCH total spike** | **52 (20-138)** | **107 (57-529)** | **167 (75-284)** | **312 (189-645)** |
| **Oxford Immunotec Panel 1+2** | **12 (6-42)** | **34 (27-70)** | **28 (16-64)** | **54 (32-230)** |
| **Oxford Immunotec Panel 14** | **20 (12-70)** | **30 (15-103)** | **40 (16-96)** | **86 (48-184)** |
| **PITCH M+NP** | **3 (0-15)** | **39 (21-90)** | **7 (0-18)** | **47 (22-119)** |
| **Oxford Immunotec M+NP** | **0 (0-4)** | **16 (11-22)** | **0 (0-4)** | **12 (5-20)** |

**Supplementary Table 1 |** Table showing the median and interquartile range for T cell responses to SARS-CoV-2 spike and structural proteins measured by PITCH ELISpot and Oxford Immunotec T-SPOT assays, subdivided by timepoint after vaccination and infection status. Healthcare workers received phlebotomy at 10 weeks post 1^st^ dose (1 dose + 10 weeks) and/or at 4 weeks post 2^nd^ dose (2 dose + 4 weeks). Infection status is divided into two groups: naïve and previously infected. All reported values are measured in spot forming units/10^6^ peripheral blood mononuclear cells (SFU/ 10^6^ PBMCs). IQR denotes interquartile range.
