# Supplementary material for: Comparison of two T-cell assays to evaluate T-cell responses to SARS-CoV-2 following vaccination in naïve and convalescent healthcare workers
Source: Clin Exp Immunol. 2022 May 6;209(1):90–8. doi: 10.1093/cei/uxac042 (PMC9129206; doi:10.1093/cei/uxac042)
Supplement: uxac042_suppl_Supplementary_Figure_Legends [file uxac042_suppl_supplementary_figure_legends.docx]

**Supplementary Figure 1 |** Antibody titres to SARS-CoV-2 spike and nucleocapsid protein in both naïve and previously-infected healthcare workers at 1 dose + 10 weeks and 2 dose + 4 weeks. (A) Antibody titres to SARS-CoV-2 spike protein in naïve and previously infected healthcare workers at 1 dose + 10 weeks and 2 dose + 4 weeks. (B) Antibody titres to SARS-CoV-2 nucleocapsid protein in naïve and previously infected healthcare workers at 1 dose + 10 weeks and 2 dose + 4 weeks. (A-B) Healthcare workers received phlebotomy 10 weeks post 1^st^ dose (1 dose + 10 weeks) and/or 4 weeks post 2^nd^ dose (2 dose + 4 weeks). Patients are matched for data measured by PITCH ELISpot and Oxford Immunotec T-SPOT assays. Roche assay was used to determine Antibody (Ig) titres in U/ml for anti-S and cut-off index (COI) for anti-N. At 1 dose + 10 weeks, n=22 for naïve samples and n=9 for previously-infected samples. At 2 dose + 4 weeks, n=36 for naïve samples and n=9 for previously infected samples. Kruskal-Wallis test was used to compare sample groups and determine statistical significance. Infection status at time of first vaccine, as defined by available PCR and serology data: grey symbols = naïve HCWs and red symbols = HCWs previously infected with SARS-CoV-2. Median T cell responses are stated immediately above each column and marked by a horizontal line on each column, and interquartile range is represented by error bars. The sample sizes are also indicated above each column in brackets.

**Supplementary Figure 2** | **Correlation between T cell responses and Antibody titres to SARS-CoV-2 spike and nucleocapsid protein.** T cell responses to spike are reported by 3 panels: PITCH total spike, Oxford Immunotec Panel 1+2 and Oxford Immunotec Panel 14. T cell responses to nucleocapsid protein are reported by Oxford Immunotec only. Roche assay was used to determine Antibody (Ig) titres in U/ml for anti-S and cut-off index (COI) for anti-N. (A-C) Correlation between antibody titres and T cell responses to SARS-CoV-2 spike protein measured at 1 dose + 10 weeks. n=31 (n=22 naïve, n=9 previously infected). (A) Correlation between SARS-CoV-2 spike antibody titres and T cell responses measured by PITCH total spike at 1 dose + 10 weeks. (B) Correlation between SARS-CoV-2 spike antibody titres and T cell responses measured by Oxford Immunotec Panel 1+2 at 1 dose + 10 weeks. (C) Correlation between SARS-CoV-2 spike antibody titres and T cell responses measured by Oxford Immunotec Panel 14 at 1 dose + 10 weeks.(D) Correlation between SARS-CoV-2 nucleocapsid antibody titres and T cell responses measured by Oxford Immunotec at 1 dose + 10 weeks. n=31 (n=22 naïve, n=9 previously infected) (E-G) Correlation between antibody titres and T cell responses to SARS-CoV-2 spike protein measured at 2 dose + 4 weeks. n=45 (n=36 naïve, n=9 previously infected). (E) Correlation between SARS-CoV-2 spike antibody titres and T cell responses measured by PITCH total spike at 2 dose + 4 weeks. (F) Correlation between SARS-CoV-2 spike antibody titres and T cell responses measured by Oxford Immunotec Panel 1+2 at 2 dose + 4 weeks. (G) Correlation between SARS-CoV-2 spike antibody titres and T cell responses measured by Oxford Immunotec Panel 14 at 2 dose + 4 weeks. (H) Correlation between SARS-CoV-2 nucleocapsid antibody titres and T cell responses measured by Oxford Immunotec at 2 dose + 4 weeks. n=45 (n=36 naïve, n=9 previously infected). Spearman’s r correlation was performed and two-tailed p values reported (alpha = 0.05). Infection status at time of first vaccine, as defined by available PCR and serology data: grey symbols = naïve HCWs and red symbols = HCWs previously infected with SARS-CoV-2.

**Supplementary Figure 3 | T cell responses to SARS-CoV-2 spike and structural proteins measured at baseline (pre-vaccination) by in-house PITCH ELISpot and Oxford Immunotec T-SPOT assay.** Healthcare workers received phlebotomy prior to 1^st^ dose vaccination (baseline). Patients here are not matched across PITCH panels and Oxford Immunotec panels. For PITCH total spike and M+NP participants, data is matched. n=39 naïve and n=25 previously infected with SARS-CoV-2. For Oxford Immunotec Panels 1+2, 14 and M+NP, data is matched. n=21 naïve (no previously infected participants available). Grey symbols indicate naïve patients and red symbols indicate patients previously infected with SARS-CoV-2, as defined by available PCR and serology. Median T cell responses are stated immediately above each column and marked by a horizontal line on each column, and interquartile range is represented by error bars. This dataset is separate from that included in Table 1. Median age is 32 years (range: 22-72 years) with 35% and 65% of the cohort being male and female, respectively.
